# Acute Lung Injury Induced by Hyperbaric Oxygen or Other External Factors, with a Focus on Exosomes

**DOI:** 10.3390/ijms27020836

**Published:** 2026-01-14

**Authors:** Jing Shi, Houyu Zhao, Chenyang Yan, Ping Zhu, Qi Zhu, Wei Ding, Longfei Wang, Yunpeng Zhao, Yue Wang, Yiqun Fang

**Affiliations:** 1 Naval Medical Center, Naval Medical University, Shanghai 200433, China; fdshijing_sj@126.com (J.S.); zhaohouyuecho@163.com (H.Z.); imzhuqi@163.com (Q.Z.); 13255589299@163.com (W.D.); wanglongfeittxn@163.com (L.W.); 2 National Key Laboratory of Immunity and Inflammation, Shanghai 200433, China; 3 Translational Medical Research Center, Naval Medical University, Shanghai 200433, China; wjczhuafanwang@163.com (C.Y.); jane124600@163.com (Y.Z.); 4 School of Life Sciences and Technology, Tongji University, Shanghai 200092, China; zhupingppzz@163.com; 5 Shanghai Institute of Stem Cell Research and Clinical Translation, Shanghai 200092, China; 6 Shanghai Key Laboratory of Cell Engineering, Shanghai 200433, China

**Keywords:** military medicine, exosomes, acute lung injury, hyperbaric oxygen, lung protection

## Abstract

Acute lung injury (ALI) is in part precipitated by hyperbaric oxygen or other mechanical insults. It constitutes the fundamental pathological process underlying acute respiratory distress syndrome (ARDS). The manifestation of the condition is characterized by an uncontrolled inflammatory response and alveolar edema, consequent to the disruption of the alveolar–capillary barrier. This phenomenon is associated with elevated morbidity and mortality rates. The current therapeutic interventions for ALI are not well researched or articulated. However, recent studies have indicated that stem cells may possess therapeutic potential in the context of ALI. The present study demonstrates that these exosome preparations have the capacity to significantly ameliorate radiographic findings, histological parameters, and vascular permeability in murine models of ALI. Concurrently, they attenuate the inflammatory response to a certain extent. The present review commences with an examination of the pathogenic mechanisms and manifestations of pulmonary injury induced by hyperbaric oxygen or other external factors. The subsequent sections of the text provide detailed accounts of the latest advances in exosome-based therapies for mitigating such injury, including their mechanisms of action and future translational prospects. While exosome-based treatments have demonstrated considerable advancement in preclinical research, numerous challenges must be surmounted before their widespread implementation in clinical settings can be realized, underscoring the necessity for sustained research in this domain.

## 1. Introduction

As demonstrated in the extant literature, oxygen is an element indispensable to human life [1,2,3]. In the contemporary era, oxygen has a ubiquitous presence in everyday life and is also extensively utilized in the medical and technological sectors. This extensive utilization has enabled its application in the development of cures and medical experimentation [4,5]. Although oxygen is essential for sustaining life, and thus must be inhaled in sufficient quantities on a daily basis, there are circumstances, such as diving or residing in high-altitude environments, in which oxygen levels can change drastically in a short time. In such cases, oxygen may exert pathophysiological toxicity on the human body [6,7]. The manifestation of central nervous system (CNS) toxicity occurs within a time frame ranging from minutes to hours in instances where oxygen partial pressure (pO_2_) surpasses the threshold of 150–160 kPa [8,9]. Conversely, pulmonary toxicity may manifest after prolonged exposure to pO_2_ levels exceeding 50 kPa, with an acceleration in the onset of symptoms observed when pO_2_ surpasses 100 kPa.

The concept of individual variability refers to the variations in the manifestation of central nervous system toxicity observed among diverse populations [10]. The Lanpier team pioneered the field of studying individual variability in carbon dioxide retention, publishing seminal research that revealed significant disparities in retention levels between divers and non-divers, with the former exhibiting notably higher concentrations [11]. It is therefore evident that individual variability is a determining factor in the assessment of the risks associated with hyperbaric oxygen exposure [12,13,14,15]. HBOT treatment has also been demonstrated to exert a measurable impact on aerobic training capacity; however, it has been demonstrated that long-term combined training is required for sustained physiological adaptation [12]. Furthermore, HBOT has been demonstrated to impact the production capacity of reactive oxygen species and the depletion of phagocytosis involving reactive oxygen species [13].

**Figure 1:**
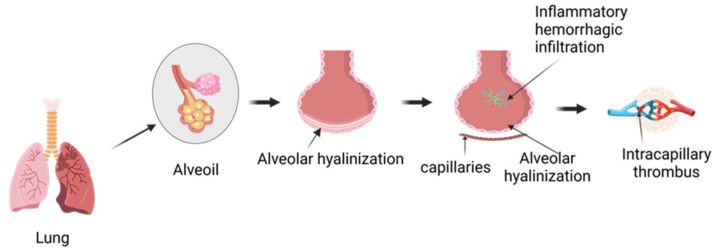
Schematic illustration of alveolar structure and alveolar–capillary barrier injury.

Hyperoxic ventilation commonly occurs in the field of pulmonary medicine, hyperbaric oxygen therapy (HBOT), and specific occupations—particularly among divers and pilots [1,9,16,17,18]. For example, underwater, pulmonary injuries caused by excessive hydrostatic pressure primarily manifest as decompression sickness (DCS), barotrauma, and Immersion Pulmonary Edema (IPE) [19]. As shown in Figure 1, following this type of high-pressure, high-oxygen lung injury, normal alveoli undergo alveolar hyalinization. With further damage, such as interstitial pulmonary edema (IPE), hemorrhage, and worsening edema, the alveoli develop additional inflammatory hemorrhagic infiltrates, and the capillaries are also damaged.

## 2. Conditions Related to Lung Injury and Oxygen Poisoning

### 2.1. Conditions and Classification of Pulmonary Oxygen Toxicity

The precise mechanism of pulmonary oxygen toxicity remains only partially elucidated, but its probable pathophysiological basis involves reactive oxygen species (ROS)-mediated alveolar damage induced by hyperoxia. Owing to their high instability, ROS disturb the lipid bilayer architecture of cell membranes and set off a cascade reaction, which in turn causes the bilayer to disintegrate and leads to alveolar epithelial cell injury and apoptosis. VOCs (volatile organic compounds) such as decane and decene may also be released during diving in oxygen-rich conditions [17,20,21]. Widespread occurrence of such damage may provoke inflammatory responses resembling ARDS. While these injuries are generally reversible after short-term hyperbaric hyperoxic exposure, prolonged exposure can progress to irreversible pathological changes characterized by pulmonary interstitial remodeling, ultimately resulting in pulmonary fibrosis and functional loss (proliferative phase), which becomes a critical risk factor for pulmonary barotrauma. Wang Yaxuan et al. indicated that recovery from a series of pulmonary injuries induced by excessive damage consistently requires preservation of alveolar epithelial barrier integrity and restoration of gas exchange function. This suggests that inadequately treated ROS-mediated lung injury may lead to irreversible impairment of pulmonary function [22,23].

In order to mitigate the risk of Potomac River (POT) pollution, a number of preventive guidelines have been established in recent decades. It has been demonstrated that there is a correlative connection between the decline in vital capacity and hyperoxic exposure through respiratory parameters. This has set the safety threshold for routine dives at 615 Unit Pulmonary Toxicity Dose per dive (corresponding to a 2% vital capacity reduction) [21,24]. In 2002, Arieli introduced the POT index and K-index, which significantly enhanced the accuracy of toxicity prediction and enabled recovery time quantification [21]. The K-index model (K = t^2^ × pO_2_^4.57^) is derived from vital capacity data obtained from pulmonary function tests. In this model, *t* denotes exposure duration (in hours) and pO_2_ represents inhaled oxygen partial pressure (in atm). However, the K-index has been shown to have limitations in repetitive diving scenarios. As stated in studies on undersea and hyperbaric medicine, it is recommended that a daily threshold of K = 120 be observed for ≤2 consecutive dive days, followed by 2 rest days. Over 5 consecutive dive days, however, this threshold should be reduced to K = 70, with subsequent 2-day rest periods. Furthermore, it is advised that daily K-values be restricted to 40–50 during serial diving without rest days [10].

### 2.2. Symptoms Associated with Decompression Sickness Lung Injury

Decompression sickness depends on dive profile (dive depth, ascent rate), dive duration, and gas type. Its core feature is the appearance of gas bubbles in the blood and organs because of quickly induced gas pressure [25]. Decompression sickness was first documented in 1841 among caisson workers. At that time, those workers showed symptoms of muscle cramps and pain [26]. However, due to the limited research conditions at the time, it was not possible to control for variables to conduct a study, so it was not certain whether such symptoms were caused by oxygen levels or other environmental factors. With the development of science, many researchers have conducted studies on this topic. To keep diving practice safe, it is suggested that recreational divers do not dive over 130 feet. They ought to ascend at a slow pace, as this lowers the risk of decompression sickness (DCI)—it stops nitrogen bubbles from expanding quickly and helps the body metabolize these bubbles more effectively [27]. DCS symptoms typically appear a few hours after a dive, accompanied by tissue damage like skin rashes, joint pain, and neurological issues [27,28,29]. In a study by DE Putra, a case report of a 25-year-old male was presented. The patient developed type 2 decompression sickness, presenting with neurological and pulmonary symptoms following a dive, including dyspnea and joint pain. After receiving positive-pressure oxygen therapy and adjunctive medication, his symptoms resolved [25]. Clinically, based on past cases and studies, DCI commonly manifests as joint pain, neurological symptoms, and rashes, often accompanied by AGE symptoms. Patients frequently exhibit stroke-like symptoms and end-organ damage following diving; surface swimmers generally do not face DCI risks. Rat experiments have confirmed that decompression sickness can also be observed and detected through muscle damage [26].

**Figure 2:**
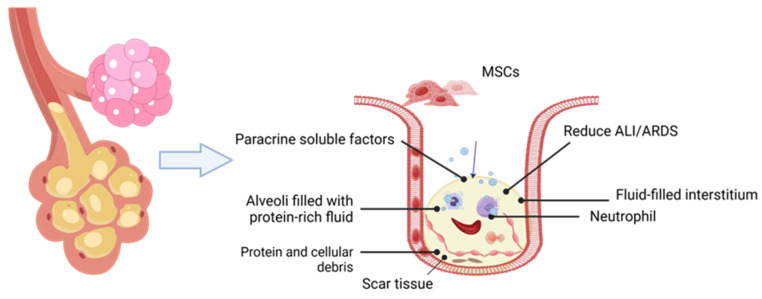
Mesenchymal stem cells (Mscs) combat ALI/ARDS lung damage via paracrine factors. The arrow above the right alveolar cells indicates the regulatory effect of soluble factors on alveolar cells.

Figure 2 shows pathological cells in ALI/ARD states on the left and right sides, with the right panel displaying magnified alveolar tissue. The tissue is filled with protein-rich fluid, accompanied by interstitial fluid accumulation, scar tissue formation, neutrophil infiltration, and protein debris accumulation. The arrow pointing to the right alveolar cell indicates the regulatory role of exosomal paracrine soluble factors on the pathological alveolar cells.

### 2.3. Pulmonary Air Pressure Injuries and Presenting Symptoms

Barotrauma is a distinct form of lung injury that arises from a rapid alteration in pressure surrounding the body. Clinically significant pulmonary barotrauma (including pneumomediastinum, pneumothorax, and life-threatening arterial gas embolism) requires only minimal pressure changes but may occur during both descent and ascent [30]. During this process, as water pressure increases, the volume of air in the lungs decreases, resulting in a reduction in lung capacity to below its smallest natural size. Initially, blood accumulates within the lung vessels. Should the compaction persist and the pressure within the lungs decrease below the external water pressure, this profound inward force has the capacity to compress the lungs, resulting in hemorrhaging [31]. Each of these diagnoses, such as subcutaneous emphysema, mediastinal emphysema, pneumothorax, pneumomediastinum, or AGE, has a different clinical presentation and can lead to diver death within minutes [32,33].

Immersion Pulmonary Edema (IPE) is another kind of lung injury disease due to recreational or military diving activities, with its first mention dating back to the 1970s [34,35]. IPE occurs when the plasma colloid osmotic pressure in the capillaries is exceeded, and it can potentially occur during diving. IPE, short for Immersion Pulmonary Edema, is an acute cardiogenic pulmonary edema syndrome that arises while individuals are swimming or diving [27,34,35,36]. Key contributors include cold water immersion combined with intense or increased ambient pressure. The pathophysiological cascade involves the following steps: (1) When the body is immersed, the generated hydrostatic pressure redirects blood toward the central part of the circulatory system. This not only boosts preload but also causes stress to pulmonary capillaries and elevates negative inspiratory pressure during the breathing process. (2) Cold immersion intensifies the constriction of peripheral blood vessels, which in turn causes a rise in both cardiac preload and afterload. (3) Exertion boosts cardiac output and systemic/pulmonary pressures, heightening capillary wall stress. (4) Potential direct capillary injury increases permeability [37,38].

### 2.4. Lung Damage Caused by Hyperbaric Oxygen Therapy

HBOT constitutes a clinical intervention conducted within a pressurized chamber containing approximately 100% oxygen. The internal pressure of the chamber exceeds atmospheric pressure at sea level, specifically exceeding 1 atmosphere absolute (ATA) [39,40,41,42]. In the context of clinical applications, it is imperative that the pressure within the chamber is maintained at a minimum of 1.4 ATA while the patient is breathing a gas mixture consisting of approximately 100% oxygen.

It is important to note that patients treated with hyperbaric oxygen may experience symptoms of central nervous system oxygen toxicity. These symptoms may include, but are not limited to, tinnitus, spasms, and even seizures. Furthermore, the therapeutic intervention may result in retrosternal discomfort, coughing, and pulmonary dysfunction. Prolonged exposure to the aforementioned therapy has the potential to culminate in the development of pulmonary fibrosis. The Unit Pulmonary Toxicity Dose (UPTD) is widely accepted as the standard metric for quantifying pulmonary oxygen exposure during HBOT and predicting the associated lung injury. It is also used to guide therapeutic protocols [43].

A study focused on exhaled volatile organic compounds (VOCs) from four divers post-heliox dives to depths > 81 m of seawater (msw) identified statistically significant changes (*p* = 0.004) in the levels of methylcyclohexane, butyl acetate, tetradecane, octane, and dodecane [21,44]. Although no abnormal respiratory symptoms or subjective discomfort was observed, the researchers hypothesized that chronic exposure to such conditions might contribute to pulmonary fibrosis. Furthermore, continuous exposure for several hours could potentially induce pulmonary oxygen toxicity (POT), with its clinical manifestations accelerating as PO_2_ increases [44].

Mediastinal emphysema, and even arterial gas embolism obstructing the coronary arteries, can result from pathological damage in severe instances of the condition [45,46]. According to clinical progression and variations in oxygenation parameters—specifically the PaO_2_/FiO_2_ ratio, with PaO_2_ representing arterial partial pressure of oxygen and FiO_2_ standing for fraction of inspired oxygen—such damage can be categorized into two types, one of which is acute lung injury (ALI). Clinically, ALI is defined by an acute onset of hypoxemia with a PaO_2_/FiO_2_ ratio between 200 and 300 mmHg, accompanied by bilateral pulmonary infiltrates, increased pulmonary permeability, and impaired gas exchange in the absence of left atrial hypertension [27,46]. Importantly, ALI and acute respiratory distress syndrome (ARDS) are considered part of the same pathological spectrum, with ARDS representing a more severe form characterized by a PaO_2_/FiO_2_ ratio persistently below 200 mmHg and more pronounced inflammatory lung injury. Consequently, a PaO_2_/FiO_2_ ratio persistently below 200 mmHg poses a significant danger to the human body. Therefore, conducting rigorous research into the process of oxygen-induced lung injury (OILI) and developing effective therapeutic interventions is of paramount importance.

## 3. Applications of Exosomes in the Treatment of ALI/ARDS

### 3.1. Introduction to Exosomes and Mesenchymal Stem Cells

Exosomes, which belong to the category of membrane vesicles and have a diameter of 30–100 nm, are formed when cytokinesis occurs [47]. Exosomes can be secreted by various types of cells, such as dendritic cells, B lymphocytes, platelets, and diverse subtypes of tumor cells [17,48,49,50,51]. These extracellular vesicles generally have a diameter ranging from 40 to 100 nm and a density of 1.13–1.19 g/mL when detected in a sucrose solution; notably, centrifugation at 100,000× *g* acts as an effective method to sediment them [52,53,54]. Yu observed that mesenchymal stem cell-derived, miRNA-encapsulating microparticles with a radius ranging from 55 to 65 nm took the form of precursors, and these were classified as exosomes [52,55,56].

Mesenchymal stem cells (MSCs) are multipotent stromal cells isolated from tissues such as bone marrow, adipose tissue, umbilical cord, and placenta. Beyond their limited differentiation capacity, MSCs exert their therapeutic effects predominantly through paracrine signaling, with exosomes constituting a major effector component of this process.

### 3.2. Exosomal Intercellular Communication Mediation

Biogenesis is initiated through endocytosis. For example, information exchange between maternal cells occurs via this process [57]. Similarly, endocytosis is the pathway through which information is exchanged among tooth germ cells [58]. The plasma membrane internalizes membrane proteins and extracellular materials to form early endosomes, establishing the structural foundation for intraluminal vesicle (ILV) assembly [59,60]. 

During this process, ILVs selectively encapsulate cytoplasmic lipids, protein, and nucleic acids [59,60,61,62,63]. Intraluminal vesicles (ILVs) exhibit two main functional tendencies: they may either fuse with lysosomes to degrade internal contents or translocate to the plasma membrane to initiate exosome secretion. Exosomes are formed when these vesicles take the latter pathway, as they are subsequently secreted into the extracellular matrix through exocytosis [17,64,65,66]. This sophisticated regulatory mechanism ensures the incorporation of diverse bioactive cargo—including various nucleic acids, tetraspanins, and heat shock proteins—within secreted exosomes [67].

**Figure 3:**
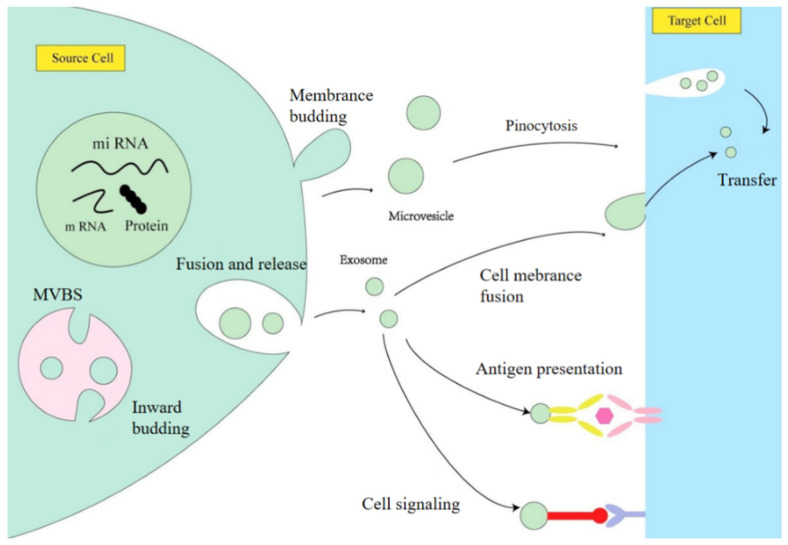
This illustration depicts exosome biogenesis, which begins with their origin from the inward budding of the multivesicular body (MVB) membrane via the endocytic pathway, and highlights their subsequent interaction with target cells. In antigen presentation, the yellow color on the right represents the ligand, while pink represents the receptor. The red color in cellular signaling denotes the ligand molecule, and blue denotes the receptor molecule (reproduced from Xie et al., *Front. Bioeng. Biotechnol.*, 2020, under CC license) [67].

As shown in Figure 3, the cytoplasm of the source cell contains miRNAs, mRNAs, and proteins that are selectively packaged into intraluminal vesicles. These form downstream of the multivesicular body (MVB). MVBs then either fuse with lysosomes to degrade their contents or fuse directly with the plasma membrane to release extracellular vesicles. These extracellularly released exosomes and vesicles can then be internalized by target cells via endocytosis or fusion with the plasma membrane, thereby participating in biological processes such as cellular signaling and antigen presentation.

Currently, exosomes are emerging as a promising therapeutic delivery pathway. Their unique vesicular structure facilitates the targeted delivery of molecular cargo—including proteins and nucleic acids—to specific cells [64]. This mechanism creates opportunities for modulating redox homeostasis and counteracting reactive oxygen species (ROS)-mediated damage, thereby offering a novel therapeutic strategy against various challenging pulmonary diseases [68,69]. Exosomes, as a cell-free therapeutic strategy, exhibit higher clinical safety and lower immunogenicity compared with cell-based therapies and are more amenable to storage, transportation, and standardization in quality control.

Despite the growing momentum in exosome research, several conceptual and experimental limitations require careful consideration. First, most current exosome-based therapies for lung diseases are derived from preclinical models, which may not fully recapitulate the complexity and heterogeneity of human acute lung injury (ALI). Second, factors such as immune responses, lung architecture, and injury repair mechanisms may constrain the therapeutic efficacy of exosomes. Finally, the inherent heterogeneity of exosomes and the lack of clinical standardization present significant challenges for commercialization. Furthermore, the regulatory mechanisms governing exosome uptake and intracellular trafficking in recipient cells remain poorly understood. Therefore, rigorous safety and contextual evaluations remain imperative before clinical translation.

Moreover, the mechanisms mediated by exosomes are highly context-dependent, exhibiting significant variations across different acute lung injury (ALI) models. In hyperoxia-induced lung injury, exosomes primarily exert protective effects by regulating oxidative stress responses, mitochondrial function, and redox balance. This protection relies heavily on the delivery of antioxidant-related small RNAs and proteins to mitigate reactive oxygen species (ROS) accumulation and epithelial cell apoptosis. In lipopolysaccharide (LPS)-induced ALI, exosome-mediated effects are primarily associated with regulating innate immune signaling and inflammatory cascades, including suppressing macrophage overactivation, inhibiting pro-inflammatory cytokine release, and modulating NF-κB and MAPK-driven inflammatory pathways. These differences indicate that the mechanisms of exosome action in ALI are not uniform but diverse.

However, the widespread clinical and commercial application of exosomes faces several challenges. First, strategies for effectively engineering or modifying exosomes to specifically target distinct cancer cell populations remain insufficiently defined. Second, exosomes may exhibit prolonged persistence in vivo, raising concerns regarding their clearance and potential off-target effects. Third, the storage conditions and long-term stability of exosomes have not yet been fully established. In addition, the abundance of exosomes may vary substantially across different cancer types [50,51]. Furthermore, the use of mesenchymal stem cells (MSCs) is widely used in medical applications because of their promising therapeutic potential and immune-modulatory characteristics [70]. The repair function of MSCs does not only rely on their intrinsic biological properties but is also influenced by paracrine signaling, which is mediated through secreted extracellular vesicles. Recent studies have indicated that MSCs, regardless of whether they are in situ or cultured in vitro, actively sense alterations in histopatho-physiological signals and integrate adaptive responses that modulate multiple components of the local immune microenvironment. This orchestrated regulation ultimately promotes tissue homeostasis, repair, and regeneration [71,72,73,74]. Over the past few years, the application of in vitro-expanded mesenchymal stem cells (MSCs) has emerged as a promising therapeutic strategy for a broad spectrum of inflammatory disorders and tissue damage [74,75,76].

Based on recent findings, exosomes derived from mesenchymal stem cells (MSCs) provide a novel and promising therapeutic approach for a range of lung disorders [24]. This observation underscores the value of MSC-Exos as a novel therapeutic option for a spectrum of lung disorders, in addition to confirming its role as a potent candidate for managing conditions from ARDS to pulmonary fibrosis. MSCs isolated from diverse sources like adipose tissue exert potent therapeutic effects by significantly improving survival, reducing inflammation, and enhancing bacterial clearance [77,78,79,80,81]. As a promising cell-free alternative to stem cell therapy, MSC-derived exosomes offer critical benefits for treating inflammatory diseases, thanks to their superior clinical safety, low immunogenicity, absence of tumorigenicity, and minimal ethical constraints [82,83,84].

MSCs exhibit considerable therapeutic efficacy in animal models of ARDS induced by endotoxins [85]. COVID-19 spread rapidly worldwide at the end of 2019, prompting an urgent search for effective therapeutic strategies for coronavirus disease. Severe acute respiratory distress syndrome (ARDS) is a major cause of mortality in patients with COVID-19 [41]. To reduce mortality, numerous pharmaceutical companies and research institutions initiated studies investigating mesenchymal stem cells (MSCs) as a potential therapeutic approach. In 2022, Xia et al. investigated exosomes derived from adipose-derived MSCs (AdMSCs) [84]. Their data demonstrated that exosomes from human AdMSCs may be effective in the treatment of inflammatory lung diseases, including COVID-19 [86]. In addition, Mesoblast reported improved clinical outcomes in patients with COVID-19 using the MSC-based product remestemcel-L [41]. MSCs’ capacity to promote tissue repair—by means of reducing alveolar leakage, suppressing inflammation, and enhancing survival rates—plays a critical role in facilitating tissue recovery. Coupled with advantageous traits like immunomodulation, low immunogenicity, and expandability, it makes them prime candidates for cellular therapeutics [87,88,89,90,91,92]. MSC-derived extracellular vesicles—characterized by a diameter ranging between 40 and 160 nm (and a mean of 100 nm)—arise from endosomal compartments and contain high levels of DNA, RNA, and proteins [93]. These vesicles primarily mediate tissue regeneration through the secretion of exosomes, bioactive molecules, and microvesicles. Released into the extracellular matrix, these vesicles participate in physiological and pathological processes. Compared to exosome therapy, MSC therapy relies on the in vivo administration of living cells. Its therapeutic effects stem not only from paracrine signaling but also from dynamic sensing of the local microenvironment and adaptive immune regulation. This characteristic endows MSCs with broader and more flexible biological functions, yet it also presents challenges such as cell viability, in vivo homing and engraftment, potential embolization risks, and complexities in production and quality control.

To date, no exosome-based therapy has demonstrated definitive efficacy in large-scale clinical trials for ALI/ARDS, and current translational outcomes remain limited or inconclusive.

### 3.3. Msc Mechanisms: Paracrine EVs and Occasional Cell Fusion

Current research indicates that the precise mechanisms of action underlying MSC therapy are not yet fully elucidated. However, several therapeutic modalities have been identified. Primarily, MSCs possess the capacity to differentiate and replace damaged cells [94]. Research indicates MSCs likely modulate vascular remodeling in other cells primarily through paracrine mechanisms [74]. For instance, absolute proof indicates that the anti-inflammatory effect of mesenchymal stem cells (MSCs) can be modulated by the secretion of extracellular vesicles (EVs) [95]. EVs are lipid bilayer-encased nanoscale particles with a cargo of a variety of RNA species, cytosolic proteins, and mitochondrial content [95,96]. Extracellular vesicles (EVs) exhibit a high degree of heterogeneity, and their composition is significantly influenced by the source of mesenchymal stem cells (MSCs), culture conditions, and the disease-specific microenvironment. This variability may account for the discrepancies observed among different studies. Extracellular vesicles (EVs) are heterogeneous populations of cells and have been recognized as vital mediators of intercellular communication [18]. Accordingly, MSC-derived EVs (MSC-EVs) possess broad-range immunomodulatory properties, which include the modulation of macrophages. Research has shown that MSC-EVs promote macrophage activation polarization from the classical pro-inflammatory (M1) to the alternative anti-inflammatory (M2) state, thus reducing the production of inflammatory cytokines [95,97,98]. However, the extent of macrophage reprogramming varies substantially across different experimental models, and the lack of standardized protocols for EV isolation methods and dosing regimens partially limits the comparability of results among studies. Such a repair process based on cellular replacement has been seen to work in renal tubular epithelial cells, epidermal keratinocytes, and endothelial cells [98,99]. With the microcirculatory rat cremaster muscle model, experiments have shown that when administered intra-arterially, MSCs are incorporated into the endothelial vascular wall [98].

Apart from this, there is direct engraftment of pulmonary MSCs into the alveolar epithelium upon their direct administration [99]. Nevertheless, this strategy is highly dependent on experimental models, and in the human in vivo environment—where inflammatory responses and oxidative stress are pronounced—the long-term survival and functional maintenance of MSCs may be substantially compromised. Though cell fusion may occur, its occurrence under disease states is considered rare. Therefore, the underlying mechanism is instead assumed to be a result of the structural aspect of lipid bilayers. Polymerase chain reaction (PCR) of organ tissues from MSC-treated patients shows that cell fusion is involved in the mediation of the curative action of MSCs [100].

A body of experimental research has been conducted in the field of mesenchymal stem cell (MSC) therapy, with scientists investigating the mechanisms underpinning the therapeutic effects of these cells. In a rat model of pulmonary hypertension, MSC suspensions or physiological saline were injected as interventions. The findings indicated that, in comparison with pre-induction levels and control groups treated with saline, rats treated with MSCs exhibited significant normalization of right ventricular pressure, attenuation of ventricular hypertrophy, and decrease in muscularization of peripheral pulmonary vessels [17,49,101]. The present study indicates that mesenchymal stem cells (MSCs) could effectively inhibit the progression of pulmonary hypertension and reverse its pathological alterations. This suggests a new therapeutic approach for hyperoxia-induced pulmonary hypertension [95].

### 3.4. Exosome-Based Therapeutics for ALI/ARDS: Mechanisms and Potential

It has been substantiated by research that MSCs hold the greatest therapeutic potential to control ALI and ARDS [87,102,103]. Paracrine signaling is mechanistically recognized as the predominant pathway through which MSCs take effect [104]. The paracrine signaling decreases alveolar epithelial permeability, suppresses cytokine response to damage, upregulates cytokine expression with anti-inflammatory properties, and reduces inflammatory cell infiltrations [105,106,107].

Importantly, MSC secretion of keratinocyte growth factor (KGF) provides a key mechanism since direct promotion of type II alveolar epithelial cell alveolar fluid clearance is afforded by this secretion [85]. Especially in *E. coli* endotoxin models of ALI, protection by KGF is accompanied by diminished pulmonary edema and attenuated lung inflammation, cementing its approach to pretreatment and prevention of ALI pathogenesis. In addition to this and through other pathways, MSCs maintain endothelial barrier integrity and epithelial homeostasis. It is also noteworthy that although such protective effects have been validated in infectious ALI animal models, their applicability to non-infectious ALI requires further investigation.

In an environment induced by lipopolysaccharide (LPS) to cause pulmonary microvascular endothelial barrier failure, hepatocyte growth factor (HGF) secreted by MSCs serves as a paracrine mediator to achieve restorative outcomes [85]. Due to paracrine secretion of HGF, pulmonary injury is reduced by MSCs through induction of mature dendritic cells (DCs) conversion to a tolerogenic type [105,107].

Research by Cheng et al. indicates that inhalation delivery of Lung Spheroid Cell Secretome (LSC-Sec) and Lung Spheroid Cell-derived Exosomes (LSC-Exo) is an effective therapy for pulmonary fibrosis and lung injury [98]. The therapeutic effect corresponding to this therapy is normal alveolar architecture re-establishment, collagen deposition reduction, and myofibroblast proliferation inhibition [64,108]. These research studies demonstrate that LSC-Sec and LSC-Exo can effectively reverse bleomycin and silica-induced pulmonary fibrosis. However, the therapeutic effect corresponding to exosomes with hyperbaric oxygen-induced pulmonary fibrosis is yet to be investigated [70,108,109].

Experimental evidence supports therapeutic exploitation of exosomes for idiopathic pulmonary fibrosis (IPF). Research suggests that excessive epithelial cell activation and apoptosis facilitate fibroblastic focus formation and excessive extracellular matrix deposition. Exosome secretion by injured or activated epithelial cells can induce fibrogenesis by activating and driving differentiation to myofibroblasts and stimulating collagen expression by fibroblasts. These exosomes carry profibrotic mediators—such as transforming growth factor-β (TGF-β), platelet-derived growth factor (PDGF), and certain microRNAs—and these mediators cause direct proliferation and collagen deposition by fibroblasts [65,110,111]. Exosomes can function both as disease-promoting factors and as potential therapeutic agents. To realize therapeutic exploitation of exosomes, research at present is directed towards achieving selective targeting of oxidative stress pathways involved with fibrotic lung injury. By selectively neutralizing reactive oxygen species (ROS), this approach abolishes the oxidative stress cascade, a driving force behind idiopathic pulmonary fibrosis (IPF) advancement. Further research should aim to elucidate the precise constitution of exosomes with a view to ensuring that their contents are those not only with potent efficacy to reduce oxidative stress but those precisely tailored to suit the individual patient’s requirements [64,112,113].

### 3.5. Structural Advantages of Exosomes in Medicine

The development of a new generation of biotherapeutic delivery systems is possible through the efficient encapsulation of therapeutic bioactive molecules with specific regenerative potential (e.g., growth factors, microRNAs, mRNAs) within exosomes or onto their membrane structures. In comparison to free active ingredients or traditional synthetic carriers, this exosome-based delivery strategy has been shown to significantly enhance drug biostability, targeting efficiency, and intracellular bioavailability [114,115,116]. Adipose mesenchymal stem cell-derived exosomes (AdMSC-Exos), which are characterized by a diameter ranging from 40 to 150 nm, are rich in components derived from donor stem cells, including proteins, nucleic acids, and lipids [82,117].

### 3.6. Mechanisms Governing the Action of Abundant Stem Cell Exosomes in Lung Injury Treatment

Accordingly, numerous studies have shown that exosomes derived from human adipose-derived mesenchymal stem cells (AdMSCs) exhibit the ability to enhance macrophage metabolism and immune homeostasis and can mitigate ALI to a certain extent [84,98,118]. The development of exosome-based therapies is imperative for treating acute lung injury, addressing the critical unmet need for effective treatments in conditions like coronavirus disease [82].

Some studies suggested that this mechanism may involve miRNAs targeting pyroptosis and immunomodulatory proteins conveyed by MSCs-Exo [64]. Researchers assessed mesenchymal stem cell-derived exosomes (MSCs-Exo) using a lipopolysaccharide (LPS)-induced ALI mouse model. The pathological features observed in this model included diffuse alveolar damage, disrupted architecture, thickened septa, inflammation, minimal fibrosis, and elevated serum/BALF IL-1β, IL-18, BALF LDH, and total protein (*p* < 0.05).

Peipei Liu et al. conducted research which revealed that the core mechanism underlying mesenchymal stem cell-derived exosome (MSCs-Exo) treatment for acute lung injury (ALI) involves the mitigation of inflammatory responses through the targeted inhibition of alveolar macrophage pyroptosis. The key therapeutic mechanisms encompass the following [92]:

1. Inhibition of Alveolar Macrophage Pyroptosis: In the LPS-induced ALI model, lipopolysaccharide (LPS) activates the NLRP3 inflammasome in alveolar macrophages (AMs). This results in the activation of caspase-1 and the subsequent occurrence of pyroptosis (marked by plasma membrane rupture and secretion of pro-inflammatory cytokines) [49,119,120]. MSCs-Exo have been shown to specifically suppress the caspase-1-mediated pyroptosis pathway by delivering miRNAs and immunomodulatory proteins.
2. Modulation of Pyroptosis-Associated Signaling Pathways: miRNA Targeting: High-throughput sequencing analysis identified MSCs-Exo as enriched with 30 significantly upregulated miRNAs that target and inhibit the expression of key pyroptosis-related genes.
3. Action of Immunomodulatory Proteins: Proteomic studies identified that MSCs-Exo contained abundant levels of immunomodulatory proteins and these proteins acted by being engaged with “immune response” and “stimulus response” signal pathways.

Mitigating Lung Tissue Damage and Inflammation: These activities eventually resulted in a significant decrease in acute lung injury (ALI) pathology as indicated by reduced structural damage to alveoli, diminished inflammatory cell infiltration, reduced permeation of the alveolar–capillary membrane, and a significant decrease in pro-inflammatory cytokine IL-1β and IL-18 levels, all of which were present and measurable in serum and bronchoalveolar lavage fluid (BALF) [121,122]. The administration with MSCs-Exo attenuated damage by reducing these markers and lung wet/dry ratio (*p* < 0.05), while MRC-5-Exo showed no therapeutic effect [92].

Collectively, mesenchymal stem cell-derived exosomes (MSCs-Exo) have been shown to attenuate the severity of acute lung injury (ALI), with this protective effect being mediated primarily through three core mechanisms: first, promoting macrophage metabolism; second, restoring immune homeostasis; third, activating signaling pathways associated with tissue repair [123,124]. A significant body of research has been conducted to verify the mechanisms through which these substances exert their effects. It has been demonstrated that they function by inhibiting the pyroptosis of alveolar macrophages and regulating the associated signaling pathways. Furthermore, their beneficial effects on lung tissue damage and inflammation have been elucidated. In the future, further investigation is required into the functional targets of these substances’ specific bioactive components, as well as their synergistic regulatory mechanisms. Optimization of extraction and delivery methods is also necessary in order to provide a more solid theoretical and practical basis for clinical transformation.

### 3.7. Exosomal Anti-Inflammatory Tissue Repair

Compared with whole-cell therapies, exosome-based interventions provide numerous benefits: their nanoscale size, significantly smaller than cells, which could deliver various bioactive proteins and signaling molecules, substantially reduces the risk of vascular occlusion [125,126,127]; they efficiently traverse biological barriers; they are amenable to engineering modifications in vitro; and they exhibit superior storage stability and clinical applicability [128]. As a result, exosome therapy emerges as a promising new method in the management of a spectrum of diseases [95]. Ligand binding to receptors, membrane fusion, and subsequent internalization are inherent processes to permit these nanovesicles to carry therapeutic payloads to selective target cells [129]. Alveolar epithelial damage is a critical pathological element commonly causing acute lung injury (ALI) [130]. Experiments have substantiated bone marrow mesenchymal stem cells (BMSCs) with therapeutic potential to repair damaged alveolar epithelial cells [130,131]. BMSC-derived exosomes (BMSCs-Exos), which function as key mediators of intercellular crosstalk, demonstrate functional responsiveness to hypoxic environmental conditions [132,133]. Furthermore, BMSCs-Exos can ameliorate smoke inhalation-induced lung injury [134]. The underlying mechanism is linked to the ability of microRNAs contained in BMSCs-Exos to regulate hypertensive injury in lung tissue [135]. MicroRNAs (miRNAs), a class of small non-coding RNAs, modulate gene expression by binding to specific target mRNAs; additionally, these molecules are involved in a wide range of human diseases [98,121,136]. Studies demonstrate the diagnostic value of miR-425 in hyperbaric oxygen-induced lung injury and ARDS [137]. By suppressing target gene expression, miR-425 inhibits colony-forming ability and cellular proliferation in lung adenocarcinoma [138].

The therapeutic efficacy of exosome therapy in ALI is primarily attributed to their favorable biocompatibility as nanoparticles and their enrichment with diverse proteins. Further mechanistic investigations have revealed that exosomal nanoparticles can markedly ameliorate pulmonary histopathological alterations, suppress inflammatory cytokine production, modulate macrophage polarization, enhance mitochondrial function, and facilitate intercellular communication.

Jingmin Fu and colleagues conducted research exploring the therapeutic application of Platycodon grandiflorus-derived exosome-like nanoparticles (PGLNs) for the treatment of acute lung injury (ALI) [139]. Utilizing an LPS-induced murine disease model, the researchers compared and contrasted the anti-inflammatory activity of fresh and dried Platycodon grandiflorus in the lung, employing fresh and dried PG preparations as experimental models. The results demonstrated a significant reduction in LPS-induced pulmonary inflammatory injury with both fresh and dried PG preparations, with fresh PG exhibiting superior therapeutic efficacy. This provides evidence for a clear therapeutic application for exosomal nanoparticles in the management of ALI. Immunofluorescence assays and in vitro studies further revealed that murine acute lung injury (ALI) defense by PGLNs is mechanistically obtained through modulating macrophage polarization.

Stem cell-derived exosomes (SC-Exos) also alleviate ALI symptoms by transferring mitochondrial components to improve alveolar macrophage homeostasis. Research by P. Liu et al. delineated the pathways underlying SC-Exo treatment of ALI: (1) transfer of mitochondria to macrophages enhances mitochondrial fitness via human mitochondrial DNA (mtDNA); (2) mitochondrial component transfer via exosomes improves macrophage mitochondrial function; (3) exosome-mediated mitochondrial transfer promotes the functional reprogramming in macrophages into an anti-inflammatory phenotypic phenotype. Collectively, these mechanisms significantly attenuate pneumonia and tissue damage in murine models [92].

### 3.8. Anti-Apoptotic Effects of Exosomes

Exosomes could modulate apoptosis across numerous diseases. Zhou et al. performed a targeted study focused on the kidney, and their findings demonstrated that mesenchymal stem cell-derived exosomes (MSCs-Exos) mitigate acute kidney injury (AKI) by suppressing the expression of Caspase-3 and promoting cell proliferation, which in turn attenuates oxidative stress and cellular apoptosis [121,140,141,142]. And the repair effect against lung injury is as mentioned in the previous subsection (exosomal anti-inflammatory tissue repair): MSC-derived exosomes inhibit alveolar epithelial and endothelial cell apoptosis, thereby protecting against lung injury [62]. Intratracheal injection of MSC-Exo or the MiR-21-5p antagonist agomir can ameliorate lung injury by significantly reducing pulmonary edema and dysfunction and inhibiting alveolar macrophage M1 polarization [143]. Let-7 microRNA (Let-7 miRNA) derived from menstrual blood stem cell-derived exosomes (MenSCs-Exos) mitigates pulmonary fibrosis by modulating three key biological processes: the levels of reactive oxygen species (ROS), damage to mitochondrial DNA (mtDNA), and the activation of the NLRP3 inflammasome. This mechanism thus offers a novel therapeutic strategy for the management of pulmonary fibrosis [18,144]. LncRNA-p21 might downregulate miR-181, and this, in turn, upregulates SIRT1—ultimately inhibiting epithelial cell apoptosis and alleviating lung tissue damage. This regulatory axis underscores the potential therapeutic function of lncRNA-p21 in ALI [145].

### 3.9. Exosomes for Cell Regeneration

The modulatory role of exosomes in promoting cellular regeneration has been well-established across a range of disease models in the extant literature [18,145]. It has been demonstrated that interstitial stem cells exhibit considerable therapeutic potential in the context of brain injury. These cells have been shown to mediate functional recovery and pathological improvement by fostering endogenous angiogenesis and neurogenesis, which in turn attenuates neuroinflammation [146]. Furthermore, the involvement of exosome in bone regeneration has been demonstrated. Liang et al. revealed that exosome derived from mesenchymal stem cells (MSCs) are capable of facilitating vascular and bone regeneration via activation of the AKT/mTOR pathway [18,147]. Furthermore, the therapeutic angiogenic effects of these particles have been demonstrated in fracture models. Specifically, human umbilical cord MSC-derived exosome therapy has been demonstrated to promote healing by upregulating CD9, CD63, and CD81 via a HIF-1α-mediated mechanism [148,149].

### 3.10. Inconsistencies in Exosome-Based Therapy for ALI/ARDS

Although a substantial body of evidence indicates that exosomes possess therapeutic potential in acute lung injury (ALI) and acute respiratory distress syndrome (ARDS), contradictory findings remain under different experimental conditions.

First, variations in lung injury induction methods represent a major source of inconsistency. In inflammation-dominant ALI models induced by lipopolysaccharide (LPS) or infection, mesenchymal stem cell-derived exosomes (MSC-Exos) consistently exhibit anti-inflammatory effects, restoration of alveolar barrier integrity, and promotion of tissue repair [85,92]. In contrast, in hyperoxia-induced lung injury models, where pathology is primarily driven by excessive reactive oxygen species (ROS) production, iron-dependent oxidative reactions, and mitochondrial dysfunction [2,45], the protective effects of exosomes are less consistent. Some studies report only transient antioxidant or anti-apoptotic effects, which are insufficient to prevent progressive structural damage [1,8,20].

Second, heterogeneity in exosome origin significantly influences therapeutic outcomes. MSC-Exos are typically enriched in anti-inflammatory, antioxidant, and pro-reparative microRNAs and proteins, enabling modulation of key signaling pathways such as PI3K/Akt, MAPK, and NF-κB, thereby attenuating inflammatory cascades in ALI [49,62]. Conversely, exosomes derived from injured epithelial cells, activated fibroblasts, or inflammatory cells may, under specific microenvironmental conditions, exacerbate inflammatory propagation or promote fibrotic processes [64,65,82].

Third, the lack of standardized administration strategies contributes to divergent results. Exosome delivery during the early injury phase or at the peak of inflammation tends to be more effective, whereas delayed intervention often fails to reverse disruption of the alveolar–capillary barrier [80,92]. In addition, methodological variability in exosome isolation, characterization, and dosing represents a critical technical factor underlying experimental inconsistency [68,96].

Finally, the bidirectional roles of exosomes across different disease stages and microenvironmental contexts warrant particular attention. While MSC-Exos facilitate resolution of inflammation and repair of alveolar epithelium in most ALI models [22,79,84], under conditions of sustained oxidative stress or abnormal mechanical stress, their long-term effects may be associated with pulmonary fibrosis or maladaptive repair [64,65,108].

In summary, these inconsistent findings indicate that exosome-based therapy for ALI/ARDS is not a universally applicable strategy, but rather a highly context-dependent intervention influenced by injury type, oxygen exposure levels, disease stage, and exosome source. Future studies should further elucidate the underlying mechanisms in hyperoxia-induced lung injury models, clarify ROS-driven pathological contexts, tailor experimental designs according to exosome adaptability, and promote standardization of exosome preparation and administration protocols to enable reliable clinical translation.

## 4. Exosomes Exert Lung Protection Through Multiple Signaling Pathways

### 4.1. Regulatory Mechanisms and Functions of the MAPK Pathway

The mitogen-activated protein kinase (MAPK) pathway serves as a core regulator in governing diverse cellular functions, including cell proliferation, differentiation, migration, and apoptosis, as well as a range of other cellular processes [150,151,152]. Four MAPK subpopulations in mammals, activated by phosphorylation cascades, trigger cellular responses via phosphorylated transcription factors and are involved in the pathogenesis of human diseases, including acute lung injury [153].

As demonstrated by Wang et al., the combination of exosome and microRNA-150 has been shown to have the capacity to suppress lung inflammation and permeability [154]. The exposure of lipopolysaccharide (LPS) to lung tissue resulted in elevated levels of total cells, neutrophils, and macrophages. Concurrently, increased concentrations of pro-inflammatory cytokines (TNF-α, IL-6, IL-1β) and total protein were observed. Furthermore, a higher dry/wet lung weight ratio was recorded. It is noteworthy that these LPS-mediated elevations were reduced following treatment with exosome and miR-150. This finding, in turn, indicates that exosome and microRNA-150 can significantly mitigate lung inflammation and the impairment of vascular permeability [64,155].

### 4.2. NF-κB Signaling Pathway Involved in Lung Protection

The nuclear factor kappa B (NF-κB) signaling pathway comprises core transcription factors that mediate immune and inflammatory responses, and it plays an indispensable role in maintaining the host’s normal physiological functions [64,98,156]. Diverse stimuli—including infection and exposure to pro-inflammatory cytokines—are capable of activating nuclear factor kappa B (NF-κB), thereby enhancing the expression of multiple inflammatory factors [18,157]. Mesenchymal stem cell-isolated exosomes (MSCs-Exos) suppress the activation of nuclear factor kappa B (NF-κB) and reduce the expression of pro-inflammatory cytokines; this dual regulatory effect, in turn, restrains inflammatory responses and cellular apoptosis [158,159,160].

### 4.3. PI3K-AKT Signaling Pathway Contributes to Lung Protection

The phosphatidylinositol 3-kinase (PI3K)-protein kinase B (AKT) signaling pathway is a core cellular signaling system that governs critical cellular processes, including cell survival, growth, proliferation, metastasis and metabolic activity. There is a strong association between this pathway and a broad spectrum of human cancers [64,151,161,162,163]. Chronic obstructive pulmonary disease (COPD) is a chronic respiratory disorder primarily characterized by persistent airflow limitation, and it arises predominantly from the inhalation of hazardous particles—specifically those derived from cigarette smoke, ambient air pollution, and biomass fuels [49,151,164]. Bronchiectasis, defined as chronic inflammation of the small airways, has been identified as a pivotal factor in the progression of chronic obstructive pulmonary disease (COPD). Specifically, Lee and his research team demonstrated that enhanced PI3K signaling exhibits a correlation with sustained inflammation in patients diagnosed with COPD [165,166]. Zhang et al. also observed that alveolar macrophages from murine models of COPD exhibited increased PI3K signaling, which resulted in a significant increase in pro-inflammatory cytokines, including TNF-α, IL-1β, and IL-6. This, in turn, led to the progression of the inflammatory reaction [18]. It can thus be posited that PTEN activation may represent a potential therapeutic strategy for the prevention of the progression of chronic obstructive pulmonary disease (COPD) [18,167].

### 4.4. Hippo-YAP Signaling Contributes to Lung Protection

Normal alveolar development and repair processes are highly dependent on the Hippo/YAP signaling pathway, which plays a pivotal regulatory role in these processes [65]. Core Hippo components—mammalian Ste20-like 1 and 2 kinases (MST1/MST2), alternatively designated as Serine-Threonine kinases 4 and 3 (STK4/STK3)—are integral to the Hippo/YAP pathway; these components interact with Salvador (SAV1) to phosphorylate large tumor suppressor kinases (LATS) [64,65,168]. Subsequently, the large tumor suppressor kinases LATS1/2 mediate the phosphorylation of YAP (an effector protein) and its cognate TAZ, which in turn inhibits their translocation into the nucleus [64,168]. During lung epithelial development, all these pathways are activated, whereas in fibrosis, they undergo dysregulation. Given that these pathways hardly act independently, understanding their intricate interactions could be crucial for determining therapeutic options for this disease. Findings from Nantie et al. further indicate that YAP signaling may be a key regulator of AT1 cell fate determination in the developing lung [169]. It has been demonstrated that in idiopathic pulmonary fibrosis (IPF), alveolar epithelial cells and activated fibroblasts exhibit aberrant YAP/TAZ activation, which is linked to increased fibrotic remodeling—this pathway thus receives mounting attention as a target for antifibrotic interventions [65].

**Figure 4:**
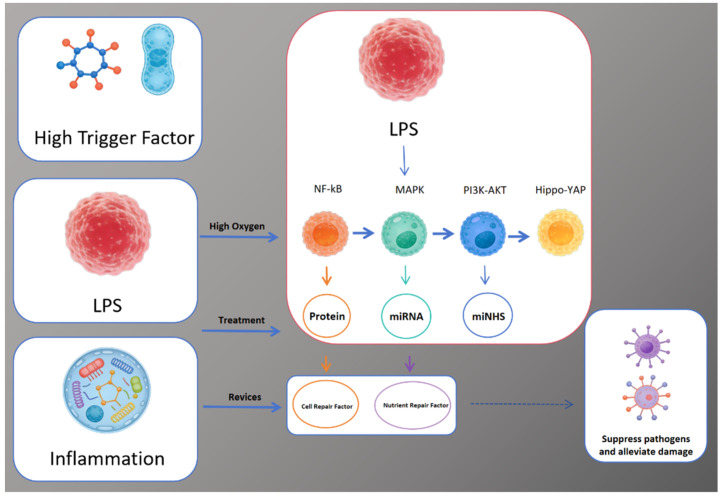
LPS-mediated signaling pathways: from trigger factors to cellular repair and pathogen suppression.

In conclusion, the regulatory network centered on lipopolysaccharide (LPS) is shown in Figure 4:

1. LPS acts as a high trigger factor (alongside other factors) to initiate responses;
2. Under high oxygen and treatment conditions, LPS activates key signaling pathways (NF-κB, MAPK, PI3K-AKT, Hippo-YAP), inducing the production of proteins, miRNAs, and mNHS;
3. These molecules further regulate cell repair factors and nutrient repair factors, which ultimately contribute to suppressing pathogens, alleviating damage, and modulating inflammation.

NF-κB, MAPK, PI3K-AKT, and Hippo/YAP interactions constitute a complex mechanism, only partially depicted in the diagram. A more comprehensive elucidation of the complete mechanism remains to be fully elucidated.

## 5. Conclusions

MSC-derived exosomes (MSC-Exos) represent an emerging experimental strategy that shows therapeutic potential in preclinical models of oxygen-related lung injury. These nanovesicles mitigate injury through multimodal mechanisms: (1) suppression of NLRP3 inflammasome-driven pyroptosis in alveolar macrophages via miRNA/protein cargo; (2) reprogramming of macrophage polarization toward anti-inflammatory M2 phenotypes; (3) restoration of cellular homeostasis through mitochondrial component transfer; and (4) paracrine delivery of regenerative factors (e.g., KGF, HGF) to enhance epithelial/endothelial repair. Crucially, MSC-Exos demonstrate superior biocompatibility, targeted biodistribution, and reduced immunogenic risk compared to whole-cell therapies.

Despite promising preclinical findings in ALI, ARDS, and fibrosis models, clinical translation of exosomes continues to face key challenges: the standardization of exosome isolation, the precise engineering of cargo, and the establishment of scalable manufacturing systems. Future research must prioritize elucidating cargo pathway specificity (e.g., ROS-neutralizing miRNAs), validating efficacy in hyperoxia-induced fibrosis models, and advancing targeted delivery systems for human applications. By addressing these obstacles, exosome-based therapies will be able to realize their role as frontline interventions for oxygen-related pulmonary injuries, as their broader application prospects are opened up.

## Data Availability

No new data were created or analyzed in this study. Data sharing is not applicable to this article.
